# The Danger of Using Chloral and Opiates Alternately

**Published:** 1878-03-20

**Authors:** A. R. Kilpatrick

**Affiliations:** Navasota, Texas


### THE DANGER OF USING CHLORAL AND OPIATES ALTERNATELY.

By A. R. Kilpatrick, M.D.

Having seen in the February number of the Southern Medical Record, an article by S. S. Shields, M. D., detailing his experience in the use of chloral hydrate before or after the administration of preparations of opium, and his request to hear from other practitioners on the subject, I improve the opportunity by writing this answer. I have long known the very same alarming effects and action of these medicines, as detailed by Dr. Shields. If he had seen the cases I have, he could not have given a better description than the one in the Record. Repetition is useless on this occasion. But in the treatment of the cases here, no battery was used, yet the patients recovered. In addition to the frictions, stimulating liniments, etc., the patients' heads were lowered and the blood made to gravitate to the brain, which seemed to produce the most decided beneficial impression. Also their mouths were opened and tongues depressed, while artificial or mechanical respiration was kept up.

My experience is altogether such as to disallow the use of chloral *after* opiates have been taken, or *vice versa*. There seems to be no bad effects where they are given *together* at the same time, but rather a more pleasant and certain soothing and hypnotic effect. Why this is so, is left for further experience to ascertain. Seeing the alarming effects of chloral, has caused practitioners here to use it with caution, or, as some do, let it alone.

Several years ago, I heard of a physician of considerable celebrity, using it in preference to chloroform when about to operate on a young man for strabismus. The physicians who were invited to assist in the operation, tried to persuade him to use chloroform, but he persisted, because he said he had less fear of the chloral, and had used it repeatedly in surgical operations.

The patient was excited and the operator kept giving chloral in unknown quantities, when the anæsthetic effects were suddenly developed in a most alarming manner; and the operator, being a pious man, in his alarm and trepidation, proposed they should all join him in prayer; but one of the assistants said, "it was no time to pray, but work like—— to save the boy's life!" And by energetic measures he was saved—though as by fire.

Not many months after that, the same party was about to operate for fistula in ano, and insisted on using chloral, although the patient plead and remonstrated; he gave it in very large, unmeasured quantities, and the recuperative energies of the man, who was past the prime of life, were insufficient to save him, although all was done which could be, as no preparation had been made to combat any wrong doing of the medicine, and the patient died before the operation was begun. As Dr. Shields said, the alarming condition of the patient supervenes suddenly, unexpectedly and with the most serious gravity. It is a very uncertain medicine in its action, varying in every case, varying in the same patient in apparently the same pathological conditions. I have used chloroform since 1848, in almost every conceivable case, and have not seen any fatal results, and seldom any alarming results. Persons using chloral should be very particular in procuring a pure article, as it is probable that some of the unfavorable results arise from using an impure specimen.

Navasota, Texas.

